# Frameworks for procurement, integration, monitoring, and evaluation of artificial intelligence tools in clinical settings: A systematic review

**DOI:** 10.1371/journal.pdig.0000514

**Published:** 2024-05-29

**Authors:** Sarim Dawar Khan, Zahra Hoodbhoy, Mohummad Hassan Raza Raja, Jee Young Kim, Henry David Jeffry Hogg, Afshan Anwar Ali Manji, Freya Gulamali, Alifia Hasan, Asim Shaikh, Salma Tajuddin, Nida Saddaf Khan, Manesh R. Patel, Suresh Balu, Zainab Samad, Mark P. Sendak

**Affiliations:** 1 CITRIC Health Data Science Centre, Department of Medicine, Aga Khan University, Karachi, Pakistan; 2 Department of Paediatrics and Child Health, Aga Khan University, Karachi, Pakistan; 3 Duke Institute for Health Innovation, Duke University School of Medicine, Durham, North Carolina, United States; 4 Population Health Science Institute, Newcastle University, Newcastle upon Tyne, United Kingdom; 5 Newcastle upon Tyne Hospitals NHS Foundation Trust, Newcastle upon Tyne, United Kingdom; 6 Moorfields Eye Hospital NHS Foundation Trust, London, United Kingdom; 7 Duke Clinical Research Institute, Duke University School of Medicine, Durham, North Carolina, United States; 8 Division of Cardiology, Duke University School of Medicine, Durham, North Carolina, United States; 9 Department of Medicine, Aga Khan University, Karachi, Pakistan; Yale University, UNITED STATES

## Abstract

Research on the applications of artificial intelligence (AI) tools in medicine has increased exponentially over the last few years but its implementation in clinical practice has not seen a commensurate increase with a lack of consensus on implementing and maintaining such tools. This systematic review aims to summarize frameworks focusing on procuring, implementing, monitoring, and evaluating AI tools in clinical practice. A comprehensive literature search, following PRSIMA guidelines was performed on MEDLINE, Wiley Cochrane, Scopus, and EBSCO databases, to identify and include articles recommending practices, frameworks or guidelines for AI procurement, integration, monitoring, and evaluation. From the included articles, data regarding study aim, use of a framework, rationale of the framework, details regarding AI implementation involving procurement, integration, monitoring, and evaluation were extracted. The extracted details were then mapped on to the Donabedian Plan, Do, Study, Act cycle domains. The search yielded 17,537 unique articles, out of which 47 were evaluated for inclusion based on their full texts and 25 articles were included in the review. Common themes extracted included transparency, feasibility of operation within existing workflows, integrating into existing workflows, validation of the tool using predefined performance indicators and improving the algorithm and/or adjusting the tool to improve performance. Among the four domains (Plan, Do, Study, Act) the most common domain was Plan (84%, n = 21), followed by Study (60%, n = 15), Do (52%, n = 13), & Act (24%, n = 6). Among 172 authors, only 1 (0.6%) was from a low-income country (LIC) and 2 (1.2%) were from lower-middle-income countries (LMICs). Healthcare professionals cite the implementation of AI tools within clinical settings as challenging owing to low levels of evidence focusing on integration in the Do and Act domains. The current healthcare AI landscape calls for increased data sharing and knowledge translation to facilitate common goals and reap maximum clinical benefit.

## Author summary

The use of artificial intelligence (AI) tools has seen exponential growth in multiple industries, over the past few years. Despite this, the implementation of these tools in healthcare settings is lagging with less than 600 AI tools approved by the United States Food and Drug Administration and fewer job AI related job postings in healthcare as compared to other industries. In this systematic review, we tried to organize and synthesize data and themes from published literature regarding key aspects of AI tool implementation; namely procurement, integration, monitoring and evaluation and map the extracted themes on to the Plan-Do-Study-Act framework. We found that currently the majority of literature on AI implementation in healthcare settings focuses on the “Plan” and “Study” domains with considerably less emphasis on the “Do” and “Act” domains. This is perhaps the reason why experts currently cite the implementation of AI tools in healthcare settings as challenging. Furthermore, the current AI healthcare landscape has poor representation from low and lower-middle-income countries. To ensure, the healthcare industry is able to implement AI tool into clinical workforce, across a variety of settings globally, we call for diverse and inclusive collaborations, coupled with further research targeted on the under-investigated stages of AI implementation.

## Introduction

The use of Artificial Intelligence (AI) tools has been exponentially growing, with several applications in the healthcare industry and tremendous potential to improve health outcomes. While there has been a rapid increase in literature on the use of AI in healthcare, the implementation of AI tools is lagging in both high-income and low-income settings, compared to other industries, has been noted, with fewer than 600 Food and Drug Administration-approved AI algorithms, and even fewer being presently used in clinical settings [1–4]. The development-implementation gap has been further assessed by Goldfarb et al., using job advertisements as a surrogate marker to measure technology diffusion patterns, finding among skilled healthcare job postings between 2015–2018, 1 in 1250 postings required AI skills, comparatively lower than other skilled sectors (information technology, management, finance and insurance, manufacturing etc.) [5].

Implementation of AI tools is a multi-phase process that involves procurement, integration, monitoring, and evaluation [6,7]. Procurement involves the scouting process before integrating an AI tool, including decisions whether to build the tool or buy the tool. Integration involves deploying an AI tool and incorporating it into existing clinical workflows. Monitoring and evaluation occur post-integration and entails keeping track of tool performance metrics, determining the impact of integrating the tool, and modifying it as needed to ensure it keeps functioning at its original intended level of performance. A key barrier highlighted by healthcare leaders across the globe to AI implementation in healthcare includes a lack of a systematic approach to AI procurement, implementation, monitoring and evaluation, since the majority of research on AI in healthcare does not comprehensively explore the multiple, complex steps involved in ensuring optimal implementation [8–11].

This systematic review aims to summarize themes arising from frameworks focusing on procuring, integrating, monitoring, and evaluating AI tools in clinical practice.

## Methods

This systematic review followed the Preferred Items for Systematic Review and Meta-Analysis (PRISMA) guidelines for systematic reviews (S1 Checklist) [12]. This review is registered on PROSPERO (ID: CRD42022336899).

### Information sources and search strategy

We searched electronic databases (MEDLINE, Wiley Cochrane, Scopus, EBSCO) until June 2022. The search string contained terms that described technology, setting, framework, and implementation phase including AI tool procurement, integration, monitoring, evaluation, including standard MeSH terms. Terms that weren’t standard MeSH terms, such as “clinical setting” were added following iterative discussions. To capture papers that were methodical guidelines for AI implementation, as opposed to experiential papers, and recognizing the heterogeneous nature of “frameworks”, ranging from commentaries to complex, extensively researched models, multiple terms such as “framework”, “model” and “guidelines” were used in the search strategy, without explicit definitions with the understanding that these encompassing terms would capture all relevant literature, which would later be refined as per the inclusion and exclusion criteria. The following search string was employed on MEDLINE: ("Artificial Intelligence"[Mesh] OR "Artificial Intelligence" OR "Machine Learning") AND ("clinical setting*"[tiab] OR clinic*[tiab] OR "Hospital" OR "Ambulatory Care"[Mesh] OR "Ambulatory Care Facilities"[Mesh]) AND (framework OR model OR guidelines) AND (monitoring OR evaluation OR procurement OR integration OR maintenance) without any restrictions. Search strategy used for the other databases are described in the appendix (S1 Appendix). All search strings were designed and transformed according to the database by the lead librarian (KM) at The Aga Khan University.

### Eligibility criteria

#### Inclusion criteria

All studies focused on implementing AI tools in a clinical setting were included. AI implementation was broadly conceptualized to consist of procurement, integration, monitoring, and evaluation. There was no restriction on the types of article included.

#### Exclusion criteria

Studies published in any language besides English were excluded. Studies describing a single step of implementation (e.g., procurement) for a single AI tool that did not present a framework for implementation were not included, along with studies that discussed the experience of consumers using an AI tool as opposed to discussion on AI frameworks.

### Study selection

Retrieved articles from the systematic search were imported into EndNote Reference Manager (Version X9; Clarivate Analytics, Philadelphia, Pennsylvania) and duplicate articles were removed. All articles were screened in duplicate by two independent pairs of reviewers (AM and JH, FG and SDK). Full texts of articles were then comprehensively reviewed for inclusion based on the predetermined criteria. Due to the heterogenous nature of articles curated (including opinion pieces) a risk of bias assessment was not conducted, as an appropriate, validated tool does not exist for this purpose.

### Data extraction

Three pairs of reviewers (SK and SG, SDK and FG, HDJH and AA) independently extracted data from the selected studies by using a spreadsheet. Pairs attempted to resolve disagreements first, followed by adjudication by a third external reviewer (ZH) if needed. Data extracted comprised of the following items: name of authors, year of publication, journal of publication, country of origin, World Bank region (high-income, middle-income, low-income) for the corresponding author, study aim(s), rationale, methodology, framework novelty, and framework components. Framework component categories included procurement, integration, post-implementation monitoring and evaluation [6,7].

### Data analysis

The qualitative data were extracted and delineated into themes based on the concepts presented in each individual study. Due to the lack of risk of bias assessment, a sensitivity analysis was not conducted. Once extracted, the themes (that encompassed the four stages of implementation (procurement, integration, evaluation, and monitoring)) were then clustered into different categories through iterative discussion and agreement within the investigator team. The study team felt that while a holistic framework for AI implementation does not yet exist, there are analogous structures that are widely used in healthcare quality improvement. One of the best established structures used for iterative quality improvement is the plan-do-study-act (PDSA) method (**S1 Fig)** [13]. PDSA is commonly used for a variety of healthcare improvement efforts [14], including patient feedback systems [15] and adherence to guideline-based practices [16]. This method has four stages: plan, do, study, and act. The ‘plan’ stage identifies a change to be improved; the ‘do’ stage tests the change; the ‘study’ stage examines the success of the change and the ‘act’ stage identifies adaptations and next steps to inform a new cycle [13]. PDSA is well suited to serve as a foundation for implementing AI, because it is well understood by healthcare leaders around the globe and offers a high level of abstraction to accommodate the great breadth of relevant use cases and implementation contexts. Hence the PDSA framework was deductively chosen, and the extracted themes from the articles (irrespective of whether the original article(s) contained the PDSA framework) were then mapped onto the 4 domains of PDSA framework, with the ‘plan’ domain representing the steps required in procurement, the ‘do’ domain representing the clinical integration, the ‘study’ domain highlighting the monitoring and evaluation processes and the ‘act’ domain representing the actions taken after the monitoring and evaluation process to improve functioning of the tool. This is displayed in S1 Table.

## Results

### Baseline characteristics of included articles

**Fig 1:**
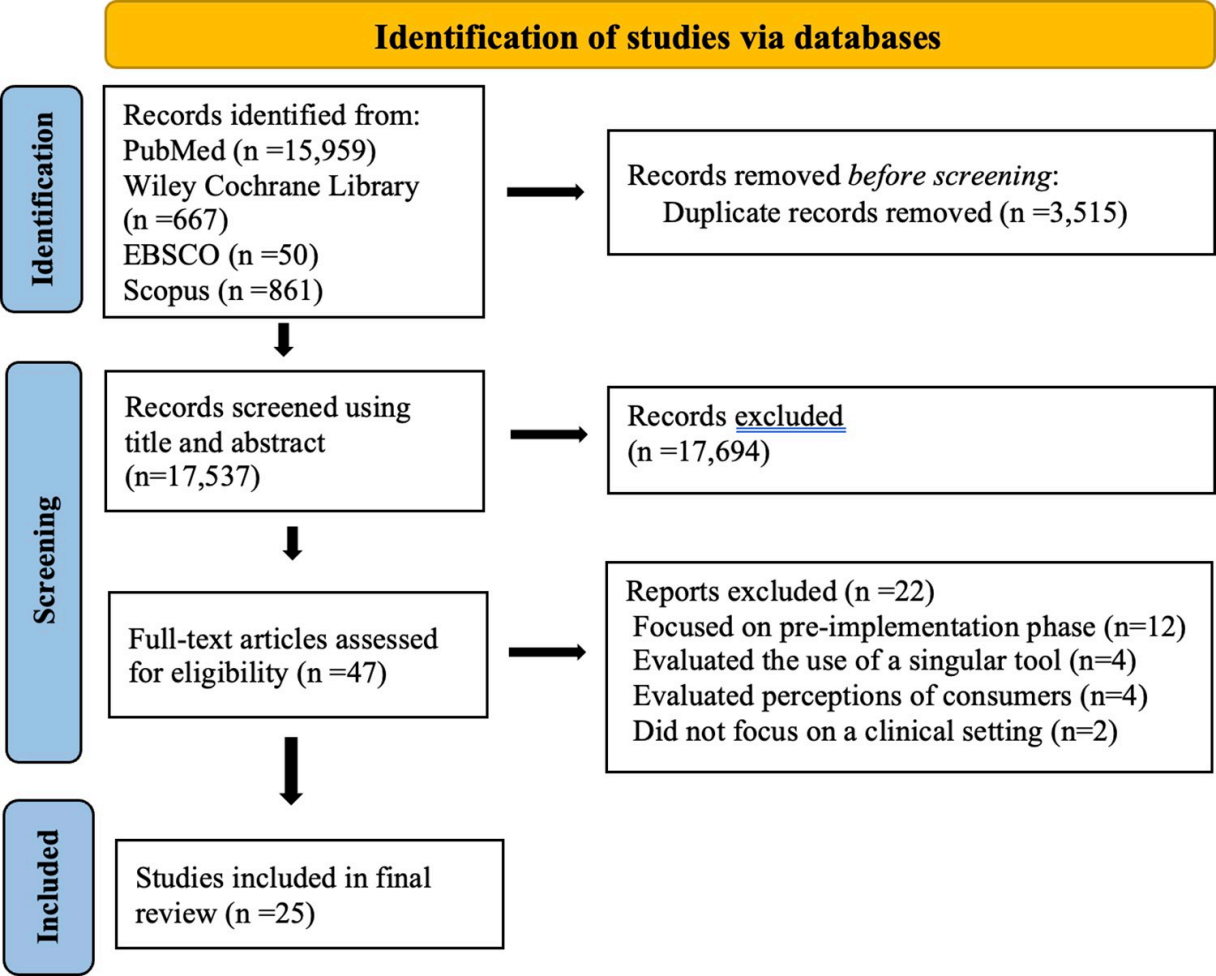
PRISMA diagram.

A total of 17,537 unique studies were returned by the search strategy, with 47 studies included after title and abstract screening for full text review. 25 studies were included in the systematic review following full-text review. 22 studies were excluded in total because they either focused on pre-implementation processes (n = 12), evaluated the use of a singular tool (n = 4), evaluated perceptions of consumers (n = 4) or did not focus on a clinical setting (n = 2). **Fig 1.** Shows the PRISMA diagram for this process. A range of articles, from narrative reviews and systematic reviews to opinion pieces and letters to the editor, were included for the review.

The year of publication of the included articles ranged from 2017 to 2022 with the most (40%, n = 10) articles being published in 2020 and the least being published in 2017 and 2018 (4%, n = 1 each). All corresponding authors of the 25 included articles (100%) originated from high-income countries with the most common country of author affiliation being United States of America (52%, n = 13), followed by the United Kingdom, Canada, and Australia (24%, n = 2 each). Among 172 authors, only 1 (0.6%) was from a low-income country (LIC)(Uganda) and 2 (1.2%) from low-middle-income country (LMIC) (India and Ghana) **(Table 1).** When stated, funding organizations included institutions in the US, Canada, the European Union and South Korea [17–24].

**Table 1:** Countries of Author Affiliations.

Economic Status of Country*	Number of Authors
High Income	167 (Top 5 contributors: United States = 68, Canada = 16, Germany = 14, Australia = 13, United Kingdom = 12)
Upper Middle Income	2 (Brazil & Costa Rica)
Lower Middle Income	2 (India & Ghana)
Low Income	1 (Uganda)

*per World Bank Income Classifications 2022–2023

### Themes

**Fig 2:**
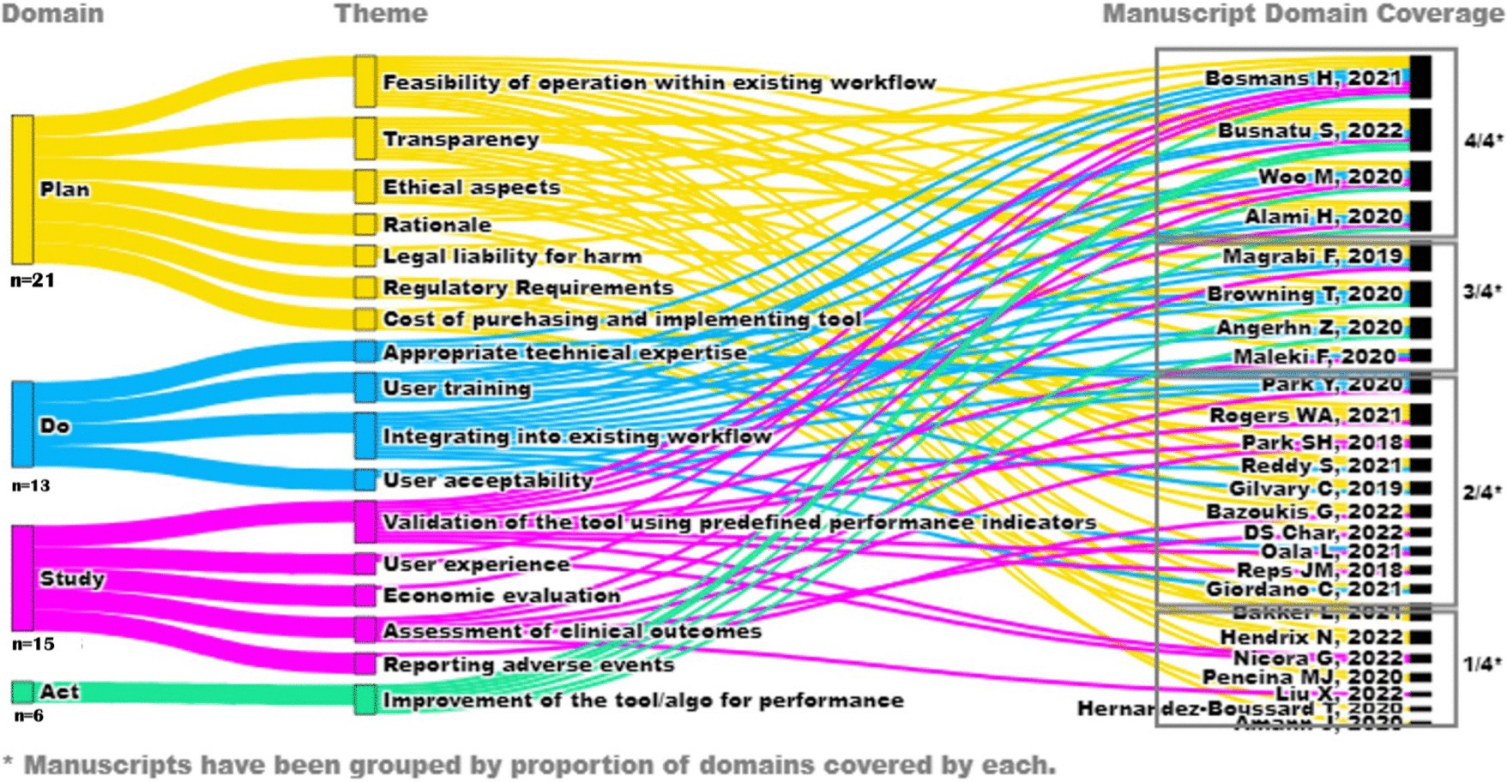
Sankey Diagram showing distribution of themes.

From the 25 included articles, a total of 17 themes were extracted, which were later mapped to respective domains. **Table 2.** Shows a summary of the distribution of themes across all the PDSA domains including a few sample quotes from eligible articles. **Fig 2.** Shows a Sankey diagram highlighting the overlap between all themes across all articles. The extracted themes are discussed below.

**Table 2:** Summary of key themes extracted from all articles.

Framework category	Theme name	Definition	Quotes
***Plan***	Rationale for using tool	The clinical rationale for starting the process of acquisition and implementation of an AI device	*“When multiple algorithms* *are available and one must be selected*, *it is* *important to evaluate any risks of data quality issues*, *and poor fit of the foundational* *data to a new situation*, *such as different* *population and morbidity patterns*.*”* [25]
	Ethical issues / bias / Contested ownership of data	Any ethical issues that are considered before deciding to consider tool acquisition	*“Veracity and deception arise in the context of AI hype*. *Honest presentation of AI in healthcare matters because both respect for autonomy and acting in patients’ best interests require a commitment to honesty*, *making it a fundamental value in the practitioner–patient relationship*. *Healthcare algorithms*, *however*, *are often developed in the context of competitive venture capitalism*, *the values of which differ from*, *and may be incompatible with*, *the values of healthcare*. *This observation suggests the need to critically evaluate new healthcare AI technologies in their social*, *legal*, *and economic contexts as well as in the clinic*. *While veracity and deception relate to the broader concepts of transparency and trustworthiness*, *both of which appear in the AI ethics literature*, *the particular issue of hype has not previously been emphasized in AI ethics frameworks*.*”* [23]
	Transparency (technical components / tool specifications / user manual, layman explanation)	Labelling entails transparency about the different aspects of AI tools so that users then make an informed purchase decision. Technical components entail a given AI tool’s model specifications. Layman explanation relates to explainability about what input variables go into the tool, how it processes the input and reaches its output.	“*A true black box is not acceptable as reproducibly is expected*. *Due to a higher accuracy with black box techniques*, *a balance is needed between the accuracy and interpretability of these methods*, *which is highly dependent on the real-world implementation setting of the tool*. *Transparency is expected in the training population*, *model functionality*, *architecture*, *risk factors and problems identified*. *Other than this there should be transparency in reporting of model performance metrics as well as the test sets and methods to derive it*.”
	Legal liability for harm	The need to identify who will be legally liable if an AI tool makes a mistake with potential adverse consequences for patient care (manufacturer, end-user, maintenance team)	*“Providing information on legal liability for harm ensuing from the use of the AI demonstrates responsibility and is required by justice*.*”* [23]
	Regulatory requirements	Ideally, AI tools need to be formally approved by the appropriate regulatory body in the country / region before being used in real-world settings. AI tools need to be assessed to ensure that they are complying with the regulatory requirements outlined by the relevant regional regulatory bodies (internal / external)	*“The regulatory environment around clinical AI may also impose costs*. *The US Food and Drug Administration has indicated that it will ask AI vendors to collect data on their algorithms’ performance and*, *potentially*, *impact on patient outcomes in the real world*. *Whereas much remains undecided on how clinical AI will be regulated*, *there is a substantial possibility that clinics will need to maintain records of how AI is used*, *which may impose data storage and processing costs*. *Another source of potential regulatory costs for clinics is that liability laws for clinicians are still nascent and*, *at present*, *expose clinicians to more liability when they use AI*. *Protections from this liability are likely to be necessary before clinicians feel empowered to use AI*.” [26]
	Cost of purchasing and implementing tool	The cost of purchasing the implementing a tool needs to be thought of and documented in advance of purchasing the tool to make sure buying and using it will be financially viable and sustainable in the long run.	*“Prices vary widely depending on customer need and most often will not include costs for hardware*, *installation*, *training*, *or maintenance*. *While the aim of these software tools is to bring about clinical or operational improvements*, *this alone may not be sufficient to drive software implementation unless direct cost savings or operational time reduction can also be attributed to the software*.*”* [27]
	Feasibility of operation within existing workflow (including data mapping)	The feasibility of integrating the AI tool within the existing clinical workflow should be assessed before purchasing it.	*“This component evaluates the usability of the AI system across different dimensions including the contextual relevance*, *and safety and ethical considerations regarding eventual deployment into clinical practice*. *It also assesses the efficiency of the system (achieving maximum productivity while working in a competent manner) as evaluated through the quality*, *adoption*, *and alignment measures*. *Utility as measured through these dimensions assesses the applicability of the AI system for the particular use case and the domain in general*.*”* [28]
***Do***	Appropriate technical expertise	The right information technology and technical support team should be involved in the integration process to ensure it goes as smoothly as possible	*“…and therefore*, *a multi-disciplinary team composed of different stakeholders with the right skills should be put in place from the start*. *This will involve*, *amongst others*, *representatives of the medical team*, *the MPE*, *the purchasing manager*, *the IT manager*, *the data scientist*, *the ethics and data protection experts*, *and the final user*.*”* [27]
	User training	The end-users (usually clinicians or nurses) need to be taught when and how to effectively use the AI tool	*“Although there is a positive attitude towards engaging AI technology in clinical practice*, *it has been reported that there is a lack of training in students and medical doctors who are supposed to work with these innovative methods*. *This aspect represents an important drawback*, *as running AI procedures by inexperienced users may lead to biased*, *subjective outcomes*. *This problem can be solved by expanding and improving medical school training in AI through familiarizing healthcare workers and taking full advantage of these emerging technologies without disregarding ethical considerations*.*”* [29]
	User acceptability	This goes in tandem with user training–the AI tool will not be a success if end-users do not find it easy to use or a worthwhile addition to their pre-existing clinical workflow.	*“AI solutions for healthcare differ from drugs or medical devices in that they are designed to affect human decision making*. *The utility of conveyed information is determined by perception*, *comprehension*, *and subsequent actions of the user*. *Hence*, *assessing the effects of AI in medicine cannot be done independently from its intended users*.*”* [30]
	Integrating into existing workflow (roles and responsibilities)	This entails incorporating the tool into existing clinical workflows so user acceptability is higher. This also comprises of assigning roles to different healthcare professionals regarding who will use, maintain, evaluate the tool over its life cycle.	*“Key concerns are whether the ML4H tool delivers utility in clinical pathways*, *how cost-effective the clinician-tool interaction is and whether it provides the desired benefits for the intended users*. *To demonstrate reliable performance*, *it is important to look beyond common machine learning performance statistics such as accuracy and to evaluate in addition whether the ML4H tool is suited to the clinical setting in which it will be used; for example*, *whether the training and test data represent patient populations that are similar to the intended use population and whether the output translates to medically meaningful parameters*.*”* [31]
***Study***	User experience	User experience comprises of the actual experience of a user using the AI tool including satisfaction/acceptance.	*“As a result*, *the prediction of the machine learning model for these instances may be often wrong*, *given that the model is applied outside its “reliable” space of work*, *leading to a decreasing trust of the final users*, *such as clinicians*. *For this reason*, *when a model is deployed in practice*, *it would be important to advise users when the model’s predictions may be unreliable*, *especially in high-stakes applications*, *including those in healthcare*. *Yet*, *reliability assessment of each machine learning prediction is still poorly addressed*.*”* [21]
	Validation of the tool using predefined performance indicators	This refers to computing predefined performance metrics like sensitivity, specificity, AUC, accuracy etc. to validate the performance of a tool in a healthcare / clinical setting.	*“Quantitative aspects of data validation*, *quality control*, *physically meaningful measures*, *parameter connections and system modelling for the future AI methods are positioned firmly in the field of the medical physics profession”*. [27]
	Cost evaluation	This entails evaluating the financial feasibility required to purchase, implement, run, maintain an AI tool including any other miscellaneous costs.	*“Member States shall ensure that the optimization includes the selection of equipment*, *the consistent production of adequate diagnostic information or therapeutic outcomes*, *the practical aspects of medical radiological procedures*, *quality assurance*, *taking into account economic and societal factors*.*”* [37]
	Assessment of clinical outcomes (while adhering to standards of care)	This refers to determining what effect the AI tool has on clinical patient outcomes such as DALYs, morbidity, mortality, length of stay, and other adverse clinical events.	*“Therefore*, *an appraisal of the effect of a diagnostic or predictive tool on patient outcome involves not just the evaluation of the tool per se*, *but an evaluation of the entire patient treatment strategy that comprises use of the tool and the subsequent chain of patient treatment steps”*. [22]
	Reporting adverse events	This refers to the mechanism of reporting and studying any potential adverse patient events or near misses that happen as a result of a selected AI tool use. Adverse event / failure reporting–post-market surveillance, user reporting of errors	*“The source organization has the responsibility for the quality and efficacy of the produced augmented intelligence algorithm*, *considering the indications and adverse effects of its use; and to provide adequate training to both physicians and personnel who will handle a specific augmented intelligence algorithm*.*”* [32]
***Act***	Improvement of the tool/algorithm for performance (sensitivity).	After all the steps in the study phase, this entails deciding if any modification in the tool itself is needed that will allow it to perform better in that specific clinical setting.	*“The support from AL/ML algorithm to continue learning and evaluating the data will shorten the time and lower the cost to provide health solution (e*.*g*., *diagnosis of a disease)*, *improve accuracy from human-alone interpretation/evaluation*, *accelerate the solution for patients as compared to traditional approach*, *and prevent disease worsening or save life”*. [33]

Seven themes were clustered and mapped to the *Plan* domain. Most articles in the Plan domain focused on the themes of feasibility of operation within existing workflows (48%, n = 12), followed by transparency (32%, n = 8) and ethical issues and bias (32%, n = 8), the cost of purchasing and implementing the tool (20%, n = 5), regulatory approval (20%, n = 5), rationale for use of AI tools (16% n = 4) and legal liability for harm (12%, n = 3). Example quotes related to each theme are captured in **Table 2**.

1) **Rationale of use of AI tools.** Frameworks highlight the need to select clinically relevant problems and identify the need for acquiring an AI tool before initiating the procurement process [27,34–36].

2) **Ethical issues and bias.** Frameworks noted that AI tools may be developed in the context of competitive venture capitalism, the values, and ethics of which often differ from, and potentially may be incompatible with, the values of the healthcare industry. While ethical considerations should occur at all stages, it is especially important, before any tool is implemented, AI tool should be critically analyzed in their social, legal, and economic domains, to ensure ethical use while fulfilling its initially intended purpose [17,18,23,27,29,32,33,37].

**3) Transparency.** Transparency of AI tools is needed to increase trust in it and ensure it is fulfilling its initially intended purpose. Black box AI tools introduce implementation challenges. Teams implementing AI must balance priorities related to accuracy and interpretability. Even without model interpretability, frameworks highlight the importance of transparency in the training population, model functionality, architecture, risk factors and outcome definition. Frameworks also recommend transparency in reporting of model performance metrics as well as the test sets and methods to derive model performance [24,25,28,29,37–40].

**4) Legal liability for harm.** There is emphasis on the legal liability that healthcare settings may face from implementing AI tools that potentially cause harm. There is a need to clarify the degree to which an AI tool developer or clinician user is responsible for potential adverse events. Relevant stakeholders involved in the whole implementation process need to be identified to know who is to be held accountable in case of an adverse event [23,25,29].

**5) Regulatory requirements:** Regulatory frameworks differ across geographies and are in flux. Regulatory decisions about AI tool adoption should be made based on proof of clinically important improvements in relevant patient outcomes [22,23,26,32,36].

**6) Cost of purchasing and implementing a tool.** Cost is an important factor to consider when deciding to implement an AI tool. The cost should be compared to the baseline standard of care without the tool. Organizations should avoid selecting AI tools that fail to create value for patients or clinicians [23,26,27,36,41].

**7) Feasibility of AI tool implementation**. A careful analysis of available computing and storage resources should be carried out to ensure sufficient resources are in place to implement a new AI tool. Some AI tools might need specialized infrastructure, particularly if they use large datasets, such as images or high frequency streaming data. Moreover, similar efforts should be made to assess the differences between the cohort on which the AI tool was trained and the patient cohort in the implementation context. It is suggested to locally validate AI tools, develop a proper adoption plan, and provide clinician users sufficient training to increase the likelihood of success [20,25,26,28,29,33,35–38,40,41].

The following four themes were clustered and mapped to the *Do* domain. Articles that were clustered in the Do domain primarily focused on integrating into clinical workflows (44%, n = 11). User training was the second most common theme (24%, n = 6), followed by appropriate technical expertise (16%, n = 4) and user acceptability (8%, n = 2). Example quotes related to each theme are captured in **Table 2**.

**1) Appropriate technical expertise**. Frameworks emphasized that the team responsible for implementing and evaluating the new AI tool should include people with different relevant expertise. Specific perspectives that should be include a machine learning expert and clinical expert (i.e. a healthcare professional who has extensive knowledge, experience, and expertise in a specific clinical area that the AI tool is being deployed for). Some frameworks suggested involving individuals with expertise across clinical and technical domains who can bridge among the different stakeholders. Inadequate representation among the team may lead to poor quality of the AI tool and patient harm due to incorrect information presented to clinician users [27,30,40,41].

**2) User training.** Frameworks highlighted the need to train clinician end users to get the maximum benefit from newly implemented AI tools, from understanding and interacting with the user interface to interpreting the outputs from the tool. A rigorous and comprehensive training plan should be executed to train the end-users with the required skillset so that they can handle high-risk patient situations [27,29,33,35,37,41].

**3) User acceptability.** Frameworks highlighted the key fact that AI models can be used in inappropriate ways that can potentially be harmful to patients. Unlike drugs, AI models do not come with that clear instructions to help users avoid inappropriate use that can lead to negative effects, hence user acceptability evaluates the how well the end users acclimatize to using the tool [25,30].

**4) Integrating into clinical workflows.** For AI tools to have clinical impact, the healthcare delivery setting and clinician users must be equipped to effectively use the tool. Healthcare delivery settings should ensure that individual clinicians are empowered to use the tool effectively [17,20,25,27,28,30,31,33,35,37,41].

Five themes were clustered and mapped to the *Study* domain. Articles that were clustered in the Study domain primarily focused on validation of the tool using predefined performance indicators (40%, n = 10). Assessment of clinical outcomes was the second most common theme (24%, n = 6), followed by user experience (8% n = 2), reporting of adverse events (4%, n = 1) and cost evaluation (4%, n = 1). Example quotes related to each theme are captured in **Table 2**.

**1) User experience.** User experience in the study domain concerned the perception of AI system outputs from different perspectives ranging from professionals to patients. It is important to look at barriers to effective use, including trust, instructions, documentation, and user training [21,27].

**2) Validation of the tool using predefined performance indicators.** Frameworks discussed many different metrics and approaches to AI tool evaluation, including metrics related to sensitivity, specificity, precision, F1 score, the area under the receiver operating curve (ROC), and calibration plots. In addition to the metrics themselves, it is important to specify how the metrics are calculated. Frameworks also discussed the importance of evaluating AI tools on local, independent datasets and potentially fine-tuning AI tools to local settings, if needed [20–23,27,29,31,35,37,39].

**3) Cost evaluation.** Frameworks discussed the importance of accounting for costs associated with installation, use, and maintenance of AI tools. A particularly important dimension of costs is burden placed on frontline clinicians and changes in time required to complete clinical duties [27].

**4) Assessment of clinical outcomes.** Frameworks highlighted the importance of determining if a given AI tool leads to an improvement in clinical patient outcomes. AI tools are unlikely to improve patient outcomes unless clinician users effectively use the tool to intervene on patients. Changes to clinical decision making should be assessed to also ensure that clinician users do not over-rely on the AI tool [18,19,22,25,30,35].

**5) Reporting adverse events.** Frameworks discussed the importance of defining processes to report adverse events / system failures to relevant regulatory agencies. Healthcare settings should agree on protocols for reporting with the AI tool developer. Software updates that address known problems should be categorized as low-risk, medium-risk or high-risk to ensure stable appropriate use at the time of updating [32].

One theme was mapped to the *Act* domain.

**1**) **Improvement of the tool/algorithm to improve performance.** Frameworks discussed the need for tailored guidance on the use of AI tools that continuously learn from new data and allowing users and sites to adjust and fine-tune model thresholds to optimize performance for local contexts. For all AI tools, continuous monitoring should be in place and there should be channels for clinician users to provide feedback to AI tool developers for necessary changes This theme was mentioned by 6 articles, with example quotes related to theme captured in **Table 2.** (24%, n = 6) [27,29,33,35,37,41].

#### Framework coverage of PDSA domains

Among the four domains (Plan, Do, Study, Act) the most common domain was Plan (84%, n = 21), followed by Study (60%, n = 15), Do (52%, n = 13), and Act (24%, n = 6). Among the 25 included frameworks, four (16%) discussed all 4 domains, four (16%) discussed only 3 domains, ten (40%) discussed only 2 domains, and seven (28%) discussed only 1 domain.

## Discussion

### Principal findings

In this systematic review, we comprehensively synthesized themes emerging from AI implementation frameworks, in healthcare, with a specific focus on the different phases of implementation. To help frame the AI implementation phases, we utilized the broadly recognizable PDSA approach. The present study found that current literature on AI implementation mainly focused on Plan and Study domains, whereas Do and Act domains were discussed less often, with a disparity in the representation of LMICs/LICs. Almost all framework authors originated from high-income countries (167 out of 172 authors, 97.1%), with the United States of America being the most represented (68 out of 172 authors, 39.5%).

### Assessment of the existing frameworks

Finding the most commonly evaluated domains to be Plan and Study is encouraging as the capacity for strategic change management has been identified as a major barrier to AI implementation in healthcare [8]. Crossnohere et al. explored 14 AI frameworks in medicine and found comparable findings to the current study where most of the frameworks focused on development and validation subthemes in each domain [42]. This focus may help to mitigate against potential risks from algorithm integration, such as dataset shift, accidental fitting of confounders and differences in performance metrics owing to generalization to new populations [43]. The need for evolving, unified regulatory mechanisms, with improved understanding of the capabilities of AI, further drives the conversation towards the initial steps of implementation [44]. This could explain why researchers often choose to focus on the Plan and Study domains much more than other features of AI tool use, since these steps can be focused on ensuring minimal adverse effect on human outcomes, before implementing the AI tool in a wider setting, especially in healthcare, where the margin of error is minimal, if not, none at all.

The most common themes in the Plan domain were assessing feasibility of model operation within existing workflows, transparency and ethical issues and bias. Researchers across context emphasized the importance of effectively integrating AI tools into clinical workflows to enable positive impacts to clinical outcomes. Similarly, there was consensus among existing frameworks to provide transparency around how models are developed and function, by understanding the internal workings of the tool to comprehend medical decisions stemming from the utilization of AI, to help drive adoption and successful roll outs of AI tools [45]. Furthermore, there is still vast public concern surrounding the ethical issues in utilizing AI tools in clinical settings [46]. The least common themes in the Plan domain were rationale for use and legal liability for harm. Without a clear problem statement and rationale for use, adoption of AI is unlikely. Unfortunately, existing frameworks do not yet emphasize the importance of deeply understanding and articulating the problem addressed by an AI tool. Similarly, the lack of emphasis placed on legal liability for harm likely stems from variable approaches to product liability and a general lack of understanding of how to attribute responsibility and accountability of product performance.

The most common theme in the Study domain was validation against predefined performance indicators. Owing to their popularity, when these tools are studied, validation and assessment for clinical outcomes compared to standard of care strategies are perhaps easier to conduct as compared to final implementation procedures. Although, validation of the tool is absolutely vital for institutions to develop clinically trustworthy decision support systems [47], it is not the sole factor responsible for ensuring that an institution commits to a tool. User experience, economic burden, and regulatory compliance are equally important, if not more important, especially in LMICs [48,49].

We found that the Do and Act phases were the least commonly discussed domains. The fact that these domains were the least focused on across medical literature may contribute to the difficulties reported in the implementation of AI tools into existing human processes and clinical settings [50]. Within the Do domain implementation challenges are not only faced in clinical applications, but also extended to other healthcare disciplines, such as the delivery of medical education, where lack of technical knowledge is often cited as the main reason for difficulties [51]. Key challenges in implementation identified previously also include logistical complications and human barriers to adoption, such as ease of use, as well as sociocultural implications [43], which remain under evaluated. These aspects of implementation potentially form the backbone of supporting the practical rollout of AI tools. However, only a small number of studies focused on user acceptability, user training, and technical expertise requirements, which are key facilitators of successful integration [52]. Furthermore, it is potentially due to the emerging nature of the field, but the Act domain was by far the least prevalent in eligible articles with only 6 articles discussing improvement of the AI tool following integration.

### Gaps in the existing frameworks

We identified that in all included articles, in the current systematic review, HICs tend to dominate the research landscape [53]. HICs have a robust and diverse funding portfolio and are home to the leading institutions that specialize in all aspects of AI [54]. The role of HICs in AI development is corroborated by existing literature, for example, three systematic reviews of randomized controlled trials (RCTs) assessing AI tools were published in 2021 and 2022 [55–57]. In total, these reviews included 95 studies published in English conducted across 29 countries. The most common settings were the United States, Netherlands, Canada, Spain, and the United Kingdom (n = 3, 3%). Other than China, the Global South is barely represented, with a single study conducted in India, a single study conducted in South America, and no studies conducted in Africa. This is mirrored by qualitative research, where a recent systematic review found among 102 eligible studies, 90 (88.2%) were from countries meeting the United Nations Development Programme’s definition of “very high human development” [58].

While LICs/LMICs have great potential to benefit from AI tools with their high disease burdens, their lack of representation puts them at a significant disadvantage in AI adoption. Because existing frameworks were developed for resource and capability rich environment, they may not be generalizable or applicable to LICs/LMICs. They considered neither severe limitations in local equipment, trained personnel, infrastructure, data protection frameworks, and public policies that these countries encounter [59] nor problems unique to these countries, such as societal acceptance [60] and physician readiness [61]. In addition, it has also been argued that AI tools should be contextually relevant and designed to fit a specific setting [44]. LICs/LMICs often have poor governance frameworks which are vital for the success of AI implementation. Governance is a key theme that is often region specific and contextual, providing a clear structure for ethical oversight and implementation processes. If development of AI is not inclusive of researchers in LICs/LMICs, it has the potential to make these regions slow adopters of technology [62].

Certain themes, which are important in terms of AI use and were expected to be extracted, were notably missing from literature. The fact that the Act domain was least discussed revealed that the existing frameworks failed to discuss when and how AI tools should be decommissioned and what needs to be considered for upgrading existing tools. Furthermore, while there is great potential to implement AI into healthcare there appears to be a disconnect between developers and end users—a missing link. Crossnohere et al. found that among the frameworks examined for the use of AI in medicine, they were least likely to offer direction with regards to engagement with relevant stakeholders and end users, to facilitate the adaption of AI [42]. Successful implementation of AI requires active collaboration between developers and end users and “facilitators” who promote this collaboration by connecting these parties [42,63]. The lack of these “facilitators” of AI technology will mean that emerging AI technology may remain confined to a minority of early adopters, with very few tools gaining widespread traction.

### Strengths, Limitations and future directions

This review has appreciable strengths and some limitations. This is the first study evaluating implementation of AI tools in clinical settings across the entirety of the medical literature using a robust search strategy. A preestablished, extensively researched framework (PDSA) was also employed for domain and theme mapping. The PDSA framework, when utilized for the distinct mapping of AI implementation procedures in the literature, has been done previously but we believe the current paper takes a different approach by mapping distinct themes of AI implementation to a modified PDSA framework [64]. The current study aimed to focus on four key concepts with regards to AI implementation, namely procurement, integration, monitoring, and evaluation. These were felt to be a comprehensive yet succinct list that describe the steps of AI implementation within healthcare settings, but by no means are meant to be an exhaustive list. As AI only becomes more dominant in healthcare, the need to continuously appraise these tools will arise and hence has important implications with regards to Quality Improvement. Limitations of the current review include the exclusion of studies published in other languages that might have allowed for the exclusion of some relevant studies and the lack of a risk of bias assessment, due to a lack of validated tools for opinion pieces. The term “decision support” was not used in the search strategy, since we were ideally looking to capture frameworks and guidelines from our search strategy on AI implementation rather than articles referring to specific decision support tools. We recognize this may have inadvertently missed some articles however, we felt the terms in the search strategy, formulated iteratively, adequately picked up the necessary articles. A significant number of articles included had an inherently high risk of bias since they are simply expert opinion, and not empirical evidence. Additionally due to the heterogeneity in language surrounding AI implementation, there was considerable difficulty conducting a literature search and some studies may not have been captured by the search strategy. Furthermore, the study only searched scientific papers from four databases, namely MEDLINE, Wiley Cochrane, Scopus, EBSCO. The current review was also not able to compare implementation processes across different countries.

In order to develop clinically applicable strategies to tackle barriers to the implementation of AI tools, we propose that future studies evaluating specific AI tools place additional importance on the specific themes within the later stages of implementation. For future research, strategies to facilitate implementation of AI tools may be developed by identifying subthemes within each PDSA domain. LIC and LMIC stakeholders can fill gaps in current frameworks and must be proactive and intentionally engaged in efforts to develop, integrate, monitor, and evaluate AI tools to ensure wider adoption and benefit globally.

## Conclusion

The existing frameworks on AI implementation largely focus on the initial stage of implementation and are generated with little input from LICs/LMICs. Healthcare professionals repeatedly cite how challenging it is to implement AI in their clinical settings with little guidance on how to do so. For future adoption of AI in healthcare, it is necessary to develop a more comprehensive and inclusive framework through engaging collaborators across the globe from different socioeconomic backgrounds and conduct additional studies that evaluate these parameters. Implementation guided by diverse and inclusive collaborations, coupled with further research targeted on under-investigated stages of AI implementation are needed before institutions can start to swiftly adopt existing tools within their clinical settings.

## Supporting information

S1 Checklist PRISMA checklist. (DOCX)

S1 Fig The PDSA cycle. (TIFF)

S1 Table Domains of the Modified PDSA framework for AI implementation. (DOCX)

S1 Appendix Search Strategy. (DOCX)
