# Supplementary figures and images for: Frameworks for procurement, integration, monitoring, and evaluation of artificial intelligence tools in clinical settings: A systematic review
Source: PLOS Digit Health. 2024 May 29;3(5):e0000514. doi: 10.1371/journal.pdig.0000514 (PMC11135672; doi:10.1371/journal.pdig.0000514)

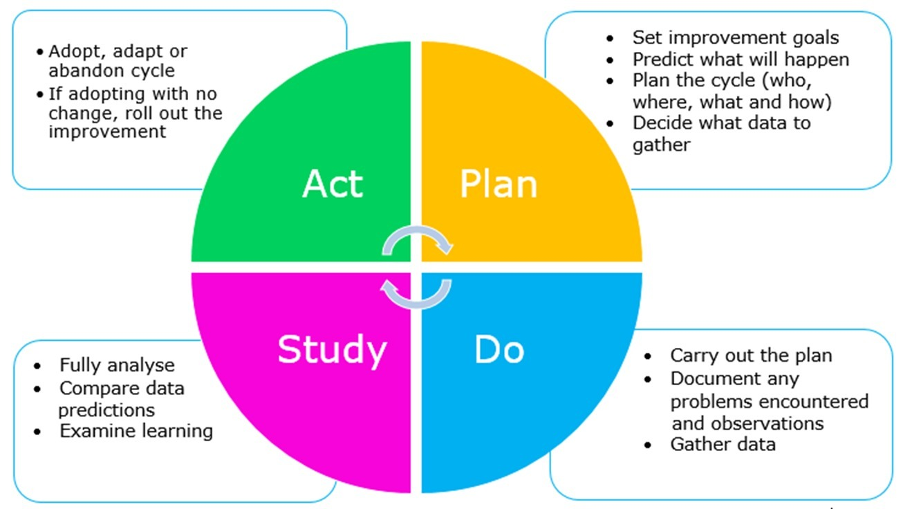

Supplement: S1 Fig — (TIFF) [file pdig.0000514.s002.tiff]
