# Supplementary material for: Frameworks for procurement, integration, monitoring, and evaluation of artificial intelligence tools in clinical settings: A systematic review
Source: PLOS Digit Health. 2024 May 29;3(5):e0000514. doi: 10.1371/journal.pdig.0000514 (PMC11135672; doi:10.1371/journal.pdig.0000514)
Supplement: S1 Table — (DOCX) [file pdig.0000514.s003.docx]

| **Domain** | **Definition** |
| --- | --- |
| Plan | A phase that illustrates the procurement process of an AI tool |
| Do | A phase that illustrates the clinical integration process |
| Study | A phase that illustrates the monitoring and evaluation process of an AI tool after integration |
| Act | A phase that illustrates any action taken after the Study phase to improve the functioning of an AI tool |
