# Supplementary material for: Frameworks for procurement, integration, monitoring, and evaluation of artificial intelligence tools in clinical settings: A systematic review
Source: PLOS Digit Health. 2024 May 29;3(5):e0000514. doi: 10.1371/journal.pdig.0000514 (PMC11135672; doi:10.1371/journal.pdig.0000514)
Supplement: S1 Appendix — (DOCX) [file pdig.0000514.s004.docx]

**S1 Appendix: Search Strategy**

PubMed NLM search strategy:

("Artificial Intelligence"[Mesh] OR "Artificial Intelligence" OR "Machine Learning") AND ("clinical setting*"[tiab] OR clinic*[tiab] OR "Hospital" OR "Ambulatory Care"[Mesh] OR "Ambulatory Care Facilities"[Mesh]) AND (framework OR model OR guidelines) AND (monitoring OR evaluation OR procurement OR integration OR maintenance))

EBSCO CINAHL Plus search strategy:

("Artificial Intelligence"[Mesh] OR "Artificial Intelligence" OR "Machine Learning") AND ("clinical setting*"[tiab] OR clinic*[tiab] OR "Hospital" OR "Ambulatory Care"[Mesh] OR "Ambulatory Care Facilities"[Mesh]) AND (framework OR model OR guidelines) AND (monitoring OR evaluation OR procurement OR integration OR maintenance))

Wiley Cochrane Library search strategy:

("Artificial Intelligence"[Mesh] OR "Artificial Intelligence" OR "Machine Learning") AND ("clinical setting*"[tiab] OR clinic*[tiab] OR "Hospital" OR "Ambulatory Care"[Mesh] OR "Ambulatory Care Facilities"[Mesh]) AND (framework OR model OR guidelines) AND (monitoring OR evaluation OR procurement OR integration OR maintenance))

SCOPUS search strategy:

("Artificial Intelligence"[Mesh] OR "Artificial Intelligence" OR "Machine Learning") AND ("clinical setting*"[tiab] OR clinic*[tiab] OR "Hospital" OR "Ambulatory Care"[Mesh] OR "Ambulatory Care Facilities"[Mesh]) AND (framework OR model OR guidelines) AND (monitoring OR evaluation OR procurement OR integration OR maintenance))
